# Evaluating the UK’s first national prescribing assessment for GPs in training using an online survey

**DOI:** 10.3399/BJGPO.2023.0044

**Published:** 2023-11-29

**Authors:** Richard Knox, Brian G Bell, Ndeshi Salema, Kim Emerson, Susan Bodgener, Jonathan Rial, Gill Gookey, Glen Swanwick, Anna Charly, Anthony J Avery

**Affiliations:** 1 School of Medicine, University of Nottingham, Nottingham, UK; 2 NIHR Greater Manchester Patient Safety Translational Research Centre, University of Manchester, Manchester, UK; 3 School of Medicine, University of Leicester, Leicester, UK; 4 Workplace Based Assessment Team, Royal College of General Practitioners, London, UK

**Keywords:** prescribing, patient safety, undergraduate education, education, general practice

## Abstract

**Background:**

GP trainees may not have experienced a systematic and comprehensive education in safe prescribing. Therefore, a self-assessment prescribing review was developed.

**Aim:**

To determine whether the assessment was feasible, had face validity, and did not disadvantage particular groups of participants.

**Design & setting:**

An online survey that evaluates the opinions of GPs in training of a prescribing assessment in the UK. All full-time UK trainees who started their final year of GP training in August 2019 undertook the prescribing assessment along with their trainers, after which they completed an online anonymous feedback questionnaire.

**Method:**

The questionnaire completed by trainees sought their opinions of the assessment, and collected ethnicity and disability data. The trainer questionnaire was similar but did not include any demographic information.

**Results:**

The questionnaire was completed by 1741 trainees and 1576 trainers. There was no evidence that ethnic group and disability were related to aspects of the review. Most of the trainees (76.4%, *n* = 1330) and trainers (82.0%, *n* = 1293) agreed or strongly agreed that the prescribing review was helpful for assessing and learning about the trainee’s prescribing. However, most participants (63.2%, *n* = 1092) took >4 hours to review their prescriptions. A majority of trainees (90.2%, *n* = 1571) reported that completing the assessment had resulted in a change in their prescribing practice.

**Conclusion:**

The majority of trainers and trainees reported that the prescribing assessment was helpful. The study was not able to assess whether there had been an actual change in practice that resulted in an error reduction.

## How this fits in

GP trainees may not have experienced a systematic and comprehensive education in safe prescribing. This study looked at the views of GP trainees and their trainers on a self-assessment of their prescribing. The majority of trainers and trainees reported that the prescribing assessment was helpful; there was no evidence of a satisfaction gap relating to ethnic group or disability. As a result, the prescribing assessment has now been completed by more than 5900 GP trainees in the UK.

## Introduction

Prescribing errors can cause significant morbidity and mortality, which healthcare organisations across the globe are committed to reducing.^1–3^ Although the General Medical Council (GMC)-funded PRACtICe study (PRevalence And Causes of prescrIbing errors in general practiCe) showed that such errors occur in about 5% of all prescriptions issued in English general practices,^4^ the REVISiT study found that the error rate for a cohort of GP trainees was almost double that (8.9%).^5^ GP trainees may not have experienced a systematic and comprehensive education in safe prescribing^6,7^ and several studies have found that junior doctors can benefit from additional training or support with prescribing skills.^6–9^

The Royal College of General Practitioners (RCGP) workplace-based assessment team (WPBA) was tasked by the GMC to instigate a meaningful assessment of prescribing for all GP trainees. Based on principles and findings from the PRACtICe and REVISiT studies, a self-assessment prescribing review was developed (which is available at: https://www.rcgp.org.uk/gp-training-and-exams/training/workplace-based-assessment-wpba/assessments).

Automated computer searches or manual consultation reviews permitted a trainee to identify the last 60 sequential prescriptions that they had issued. Trainees would then interrogate their prescriptions to ascertain if each one contained any errors or elements of suboptimal prescribing. A pre-formatted Excel spreadsheet was available for trainees to download to facilitate documentation.

An error occurred when then had been an *‘unintentional significant reduction in the probability of treatment being timely or effective; or (an) increase in the risk of harm when compared with generally accepted practice’.* ^10^ Suboptimal prescribing was defined as *‘less than ideal practice*’^4^ that did not fit the strict error definition.

Systematic interrogation took the trainees through a process of deciding if they had prescribed the correct drug for the indication, at the right dose, with appropriate dosage instructions. They would then determine if they had instigated appropriate follow-up, and documented everything appropriately. Finally, they would highlight any aspects of ‘good prescribing’ that they had identified. The interrogation process is summarised in Table 1.

**Table 1.** GP trainee prescribing review checklist

Prescribing area	Areas to consider
**Right drug**	Evidence for use in the indication Allergies Contraindications or cautions Interactions with co-prescribed medication Local and national prescribing guidelines Local formulary Social issues (for example, carers, inclusion in a monitored dosage system) Formulation Duplication or omissions in therapy Correct use of brand prescribing for safety reasons
**Right dose**	Renal or hepatic function Age and weight Local and national prescribing guidance Is the dose correct for the indication? Has increasing or reducing dosing been done appropriately? Most appropriate strength of tablet prescribed for the required dose
**Right dosage instructions**	Clear and unambiguous (avoiding 'as directed') Up to date (according to current usage or latest letters) Include route of administration or area of application Are the instructions able to be read and understood by the patient?
**Right follow-up**	Has the necessary monitoring been planned, taken, or acted on; for example, blood tests, blood pressure measurement? Has the item been placed on repeat appropriately so that it cannot be continued without a necessary review?
**Right documentation**	Is the indication for prescribing clear and relevant? If prescribing does not follow standard guidance, is the reason documented? Is the plan for any necessary monitoring or follow-up documented?
**Right review**	Where the medication has been used before, has under- or over-ordering been addressed before supplying (adherence to therapy)? Have any necessary discussions taken place before continuing medications with risks; for example, hormone replacement therapy?
**Good prescribing**	Does prescribing show that local guidelines have been referred to; for example, antimicrobial guidelines? Is the prescribing plan in the notes and thought process accurate and clear for the next clinician to follow? Clarity of advice regarding recommendations for over-the-counter medications

Trainees were able to refer to a detailed prescribing manual, which contained ‘case law’ of prescribing errors and suboptimal prescribing that had previously been identified in the PRACTiCe and REVISiT studies (which is available at https://www.rcgp.org.uk/mrcgp-exams/wpba/assessments). ‘Case law’ facilitated the decision as to whether a prescribing scenario should be classified as a particular prescribing problem.^4^ Examples of such case law can be found in Supplementary Table S1.

When an error or suboptimal prescribing was identified, trainees would reflect on why this had occurred, and consider strategies that could be adopted to avoid recurrence in future practice. Once trainees had reviewed 60 prescriptions, their trainer or supervisor would review a random subset of at least 20 of these prescriptions in order to validate their trainee’s review process. The two parties would then meet for a structured tutorial and determine what steps may be necessary to cement good prescribing practices. To ‘pass’ the prescribing assessment, trainees had to demonstrate adequate engagement with the process and undertake appropriate reflective practice.

Before the structured self-review process could be adopted as a prescribing assessment for all GPs in training, it was necessary to determine whether the assessment was feasible, had face validity, and did not disadvantage particular groups of participants. Therefore, GP trainees were engaged in a pilot of the prescribing assessment. At the end of the assessment process, trainees and trainers were invited to complete a detailed feedback questionnaire. This article reports the quantitative findings from the feedback questionnaire, which were used to support the adoption of the prescribing assessment for future GPs in training. Free-text feedback comments were also sought, which will be presented elsewhere.

This evaluation aimed to present the following:

- the number of prescribing errors and suboptimal prescribing instances reported by GP trainees;
- the impact of the prescribing assessment on trainees’ and trainers’ time;
- whether there were reported concerns around trainees with certain protected characteristics being disadvantaged by undertaking the prescribing assessment;
- feedback regarding the utility and validity of the prescribing assessment from the perspective of trainer and trainee.

## Method

All full-time UK GP trainees who started their final (ST3) year of GP training in August 2019, who would be due to complete GP training in August 2020, undertook the prescribing assessment as outlined above. All trainees and their trainers were then invited to complete a detailed online anonymous feedback questionnaire, which was hosted on the Jisc platform (https://www.onlinesurveys.ac.uk/). They were reminded to complete this survey through means of a reminder email, and at their final training review. The trainee and trainer questionnaires were not linked to ensure the anonymity of the responders.

The questionnaires were developed by the RCGP WPBA in partnership with the research team in Nottingham, and then piloted to ensure ease of understanding. The questionnaire completed by trainees collected some demographic information, and sought trainees’ opinions of the assessment. The trainer questionnaire was similar but did not include any demographic information. Both questionnaires can be found in Supplementary Table S2.

Frequency counts for the variables that assessed the trainee characteristics, the trainees’ and trainers’ evaluation of the prescribing assessment, and the outcome of the prescribing assessment were examined. Means and standard deviations (SDs) were calculated for how many prescriptions the trainee and trainer reviewed. The authors also ran correlations (rank-biserial correlation or point-biserial correlation) between the protected characteristics of the trainees (ethnic group and disability) and various aspects of the prescribing assessment, including ease of completing the assessment, time taken to complete the different stages of the prescribing review, number of prescribing errors, number of suboptimal prescribing errors, and the trainee’s helpfulness and acceptability ratings of the assessment. For suboptimal and prescribing errors, the point-biserial correlation was used, for all other outcomes, the rank-biserial correlation was used. All analyses were conducted using IBM SPSS Statistics (version 26).^11^

The evaluation did not undergo formal ethics committee review, but as it related to a potential new assessment to be undertaken by GPs in training, the process was reviewed and approved by the RCGP Assessment Team and the Committee of General Practice Education Directors (COGPED). As the assessment had not yet been evaluated, it was important to ensure that no trainee would be disadvantaged by completing the assessment; for example, an unsatisfactory outcome in this pilot cohort would not result in a trainee having to extend their training.

## Results

### Participants

The assessment was open to all GP trainees who were due to complete their GP training in August 2020 and formed a compulsory part of the training portfolio for full-time trainees. It is known that 1439 trainees completed training at this point. Accurate numbers for those who started the training year were not available. The feedback questionnaire was completed by 1741 GP trainees and 1576 GP trainers. It can therefore be assumed that the majority of eligible GP trainees completed the feedback questionnaire and at least 90% of GP trainers (*n* = 1576/1741), as some trainers may have supervised more than one trainee. The characteristics of the trainees who completed the feedback survey are summarised in Table 2. It should be noted that the percentage of trainees in the sample who were from an ethnic minority (44.3%) matches closely the percentage of ethnic minority trainees (46%) from all specialties in the UK in 2021.^12^

**Table 2.** GP trainee characteristics

GP trainees’ characteristics	Frequency, *n* (%)
Ethnic group	
White	891 (52.1)
Asian, Asian British	499 (29.2)
Black, African, Caribbean, Black British	128 (7.5)
Mixed or Multiple ethnic groups	45 (2.6)
Other ethnic group	37 (2.2)
Prefer not to say	110 (6.4)
Disability	
No	1589 (93.3)
Yes^a^	38 (2.2)
Prefer not to say	76 (4.5)
Where primary medical qualification gained	
UK	1319 (77.2)
Rest of the world	267 (15.6)
European Economic Area	74 (4.3)
Prefer not to say	49 (2.9)
Type of practice	
Urban	972 (56.8)
Rural and urban mixed	491 (28.7)
Rural	189 (11.1)
Prefer not to say	58 (3.4)
Nation of training	
England	1374 (80.6)
Scotland	182 (10.7)
Northern Ireland	55 (3.2)
Wales	54 (3.2)
Prefer not to say	40 (2.3)
Outcome 2, 3, & 5 at any point of training^b^	
No	1340 (78.3)
Yes	208 (12.2)
Prefer not to say	163 (9.5)

^a^Of those with a disability: 26 (68.4%) had dyslexia; 3 (7.9%) had dyslexia and dyspraxia; 1 (2.6%) had dyslexia, learning disability, and a mental health condition; 1 (2.6%) had a mental health condition; 5 had a physical disability (13.2%); 1 had diabetes (2.6%); and 1 had epilepsy (2.6%). ^b^Outcome 2 is 'Development of specific competencies required — additional training time not required'. Outcome 3 is 'Inadequate progress by the trainee — additional training time required'. Outcome 5 is 'Incomplete evidence presented — additional training time may be required'.

### Outcome of the prescribing assessment

As noted, trainees were expected to review 60 consecutive prescription items as part of the prescribing assessment. A total of 1739 (99.9%) trainees reported reviewing at least one prescription; the mean number of prescriptions reviewed was 59.9 (SD = 8.0). Trainers were instructed to review 20 of their trainee’s prescriptions, the mean number of prescriptions reviewed by trainers was 22.0 (SD = 7.3). Most of the trainers (*n* = 1302, 82.6%) thought that reviewing 20 of their trainee’s prescriptions was ‘*just about right*’.

The results of the assessment revealed that at least one prescribing error was uncovered by 1244 trainees (71.5%), and 1714 trainees (98.4%) found at least one incident of suboptimal prescribing. The mean number of suboptimal errors was 24.3 (SD = 19.4) and the mean number of prescribing errors was 5.0 (SD = 8.6). The mean prescribing error rate and suboptimal prescribing rate (*n* = 1739) were 8.3% and 40.6%, respectively.

Ethnic group and disability were correlated with various aspects of the prescribing assessment, including ease of completing the assessment, time taken to complete the different stages of the prescribing review, number of prescribing errors, number of suboptimal prescribing errors, and the trainee’s helpfulness and acceptability ratings of the assessment. There was no evidence that ethnic group and disability were related to aspects of the review. The correlations were small: all were <0.20.

### Evaluation of the assessment

**Figure 1.**
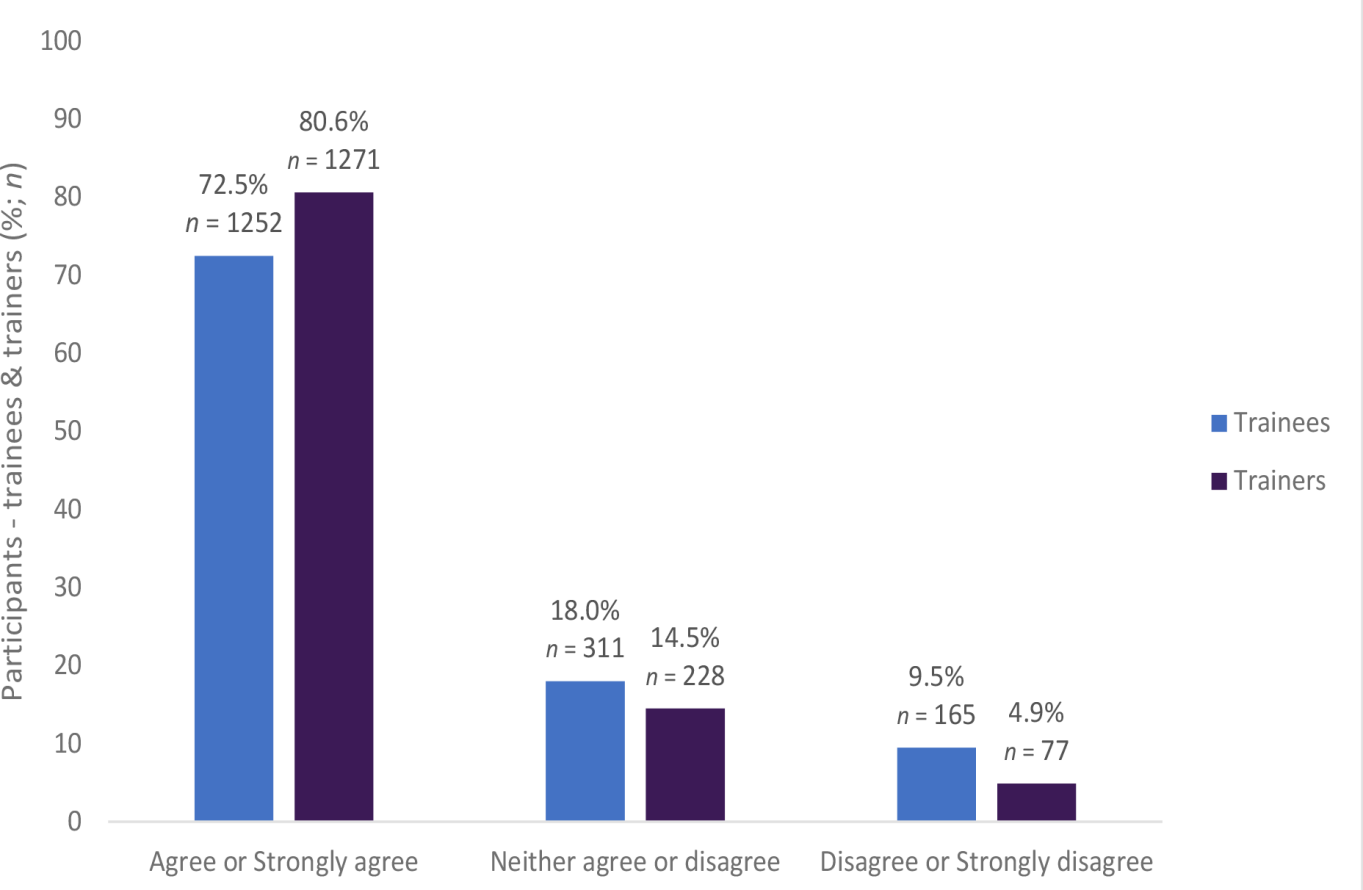
The guidance document provided sufficient information to make a decision on the nature of the errors made (trainees *n* = 1728, trainers = *n* = 1576)

**Figure 2.**
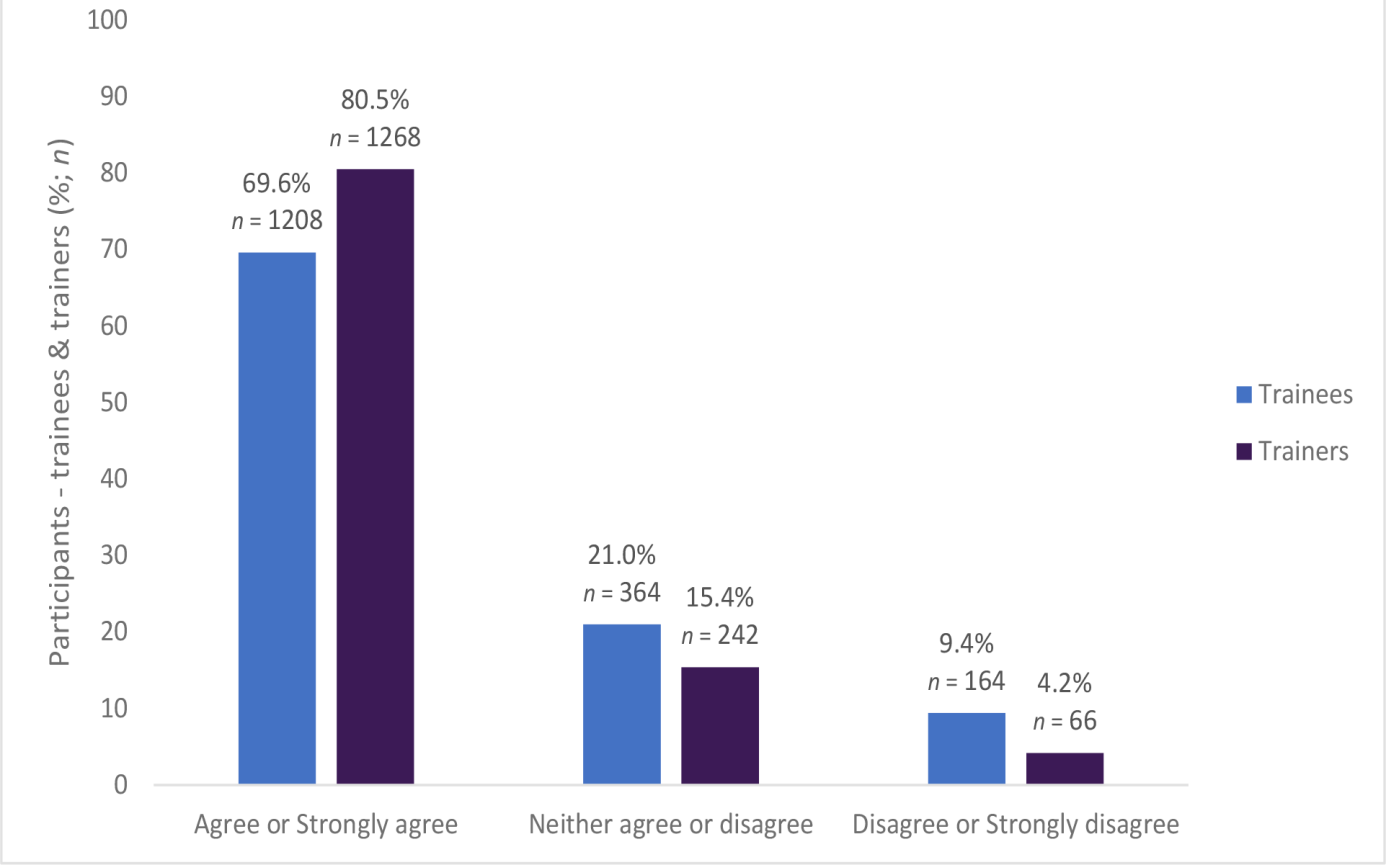
The guidance document provided sufficient information to assess examples of good prescribing (trainees *n* = 1736, trainers *n* =1576)

Most trainees (*n* = 801, 51.8%) agreed that the computer search instructions were easy to follow and complete with one quarter (*n* = 392, 25.4%) disagreeing, and 22.9% (*n* = 354) not having an opinion (trainee responses are for *n* = 1546). Most of the participants (63.2%, *n* = 1092) took >4 hours to review their prescriptions. A high percentage of trainees (72.5%, *n* = 1252) and trainers (80.6%, *n* = 1271) reported that the guidance documents provided for the assessment were suitable for helping them make decisions on the nature of the prescribing errors they had identified (see Figure 1; trainee responses are for *n* = 1728). Furthermore, most of the trainees (69.6%, *n* = 1208) and trainers (80.5%, *n* = 1268) agreed that the guidance document provided sufficient information to assess examples of good prescribing (see Figure 2; trainee responses are for *n* = 1736).

**Figure 3.**
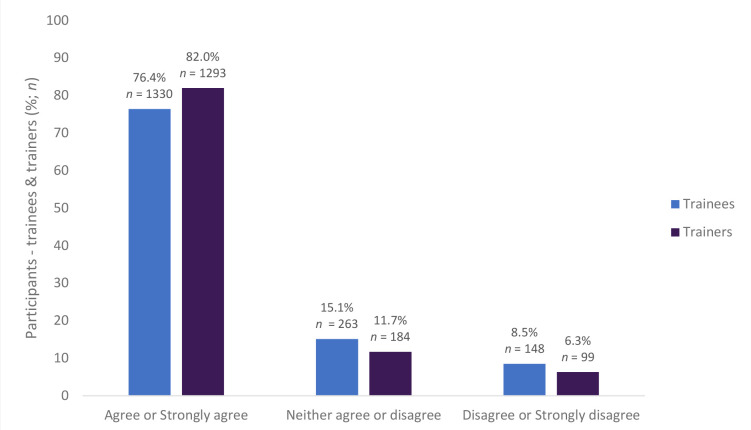
The prescribing review was helpful for trainees assessing and learning about prescribing and trainers assessing their trainee’s prescribing (trainees *n* = 1741, trainers *n* = 1576)

**Figure 4.**
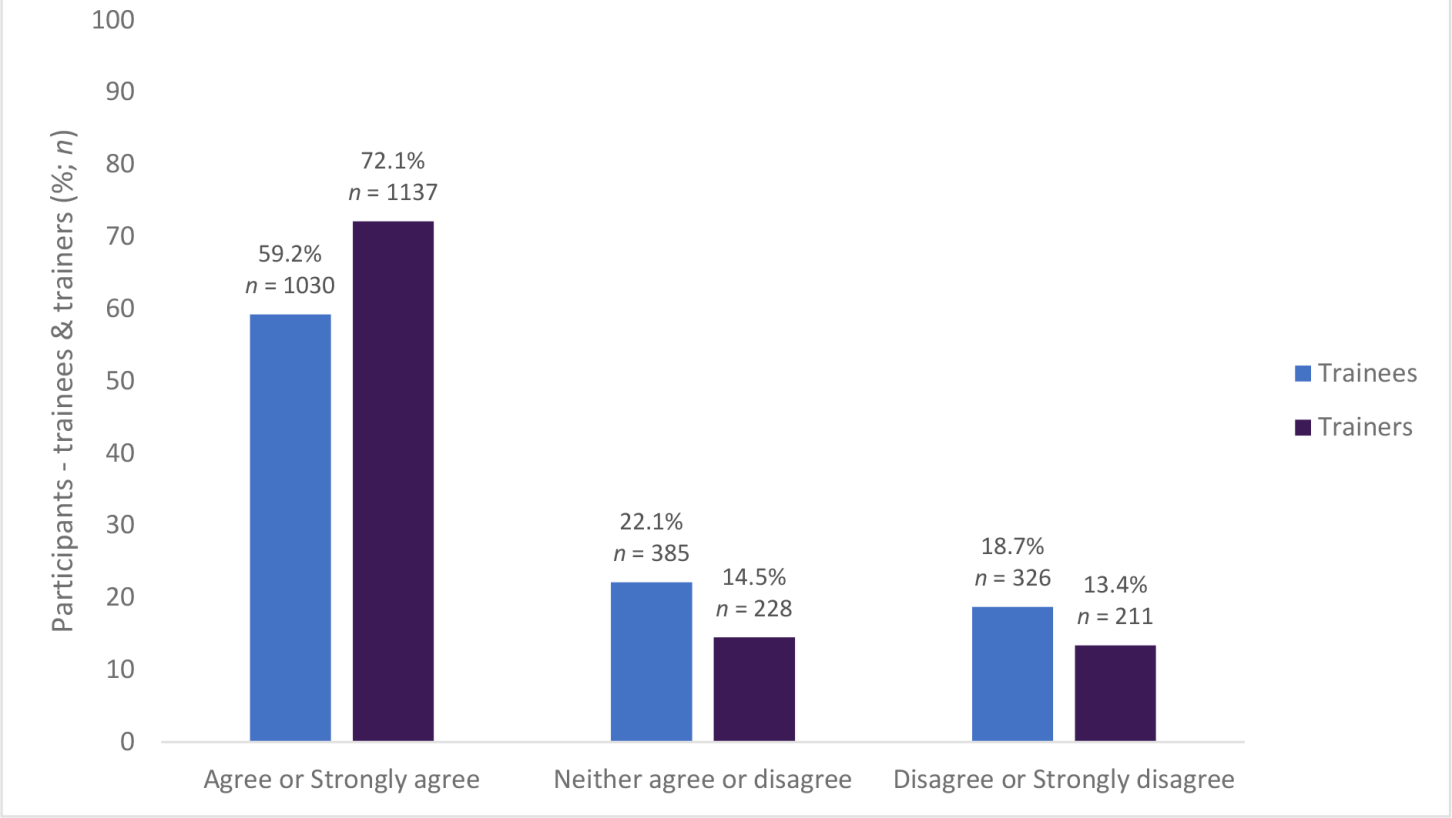
The prescribing review is an acceptable prescribing assessment (trainees *n* = 1741, trainers *n* = 1576)

Most of the trainees (76.4%, *n* = 1330) and trainers (82.0%, *n* = 1293) agreed or strongly agreed that the prescribing review was helpful for assessing and learning about the trainee’s prescribing (see Figure 3). The prescribing assessment was viewed by both trainees (59.2%, *n* = 1030) and trainers (72.1%, *n* = 1137) as an acceptable assessment (see Figure 4).

A majority of trainees 90.2% (*n* = 1571) reported that completing the assessment had resulted in a change in their prescribing practice. When trainers were asked to compare the expected level of prescribing proficiency for their trainee for their current stage of training, 93.1% (*n* = 1468) indicated that their trainee was *‘a safe, reflective GP prescriber at this point in time*’. The assessment of 6.5% of the trainers (*n* = 103), was that their trainee needed ‘*to develop specific prescribing skills as identified in the PDP*’. The remaining five trainers (0.3%) noted that their trainees need *‘to repeat the whole assessment*’.

Most of the trainers (87.6%, *n* = 1381) stated that the prescribing they reviewed covered a wide range of patient types, such as that would normally be seen in general practice. More than half of the trainers (56.4%, *n* = 889) thought that the assessment highlighted gaps and areas of improvement for the trainee that the trainer was not aware of. More than half of the trainers (62.3%, *n* = 982) mentioned that this review caused them to reflect on the training and support they would offer to future trainees.

## Discussion

### Summary

A total of 1741 trainees and 1576 trainers completed the questionnaire for the prescribing assessment. The mean error rate reported by trainees was 8.3%. There is no evidence that the self-assessment prescribing review put any group of trainees at a disadvantage. However, most of the trainees took >4 hours to review their prescriptions, which could be a considerable time burden.

The majority of trainers and trainees reported that the prescribing assessment was helpful, and that it was an acceptable way of assessing prescribing. Furthermore, trainees found the assessment was easy to complete with the vast majority reporting that completing the assessment had resulted in a change to their prescribing practice. The assessment’s utility for GP training was further supported by the fact that more than half of trainers (56.4%, *n* = 889) thought that it had highlighted areas of improvement for their trainee that they had previously been unaware of.

### Strengths and limitations

One of the strengths of this study was the large sample size, which may have been influenced by all trainees being prompted to complete the questionnaire at their final training review session. More trainees completed the questionnaire than are recorded as finishing training in August 2020. It is possible that some ST3 trainees completed the questionnaire, but for some reason were delayed from completing training when they were ‘scheduled’ to (for example, by requiring an extension to training). A small number of ST3 trainees may have started their training year after August 2019, but still completed the assessment and evaluation as if they were due to finish in August 2020. In this pilot year, the assessment was not mandated for part-time trainees, although they were encouraged to complete it. It may have been beneficial to explore perceptions of the assessment in this cohort of trainees, especially as participant gender was not recorded; female trainees are more likely to work part-time with caring responsibilities than are their male counterparts.^13^

Although the study asked both trainees and trainers to evaluate the prescribing review and assessment, there was no way to correlate these responses as they were anonymous and not linked. The study was also not able to assess whether there had been an actual change in practice that resulted in an error reduction.

### Comparison with existing literature

The mean error rate reported by trainees (8.3%) was comparable to the error rate found in the REVISiT study (8.9%).^5^ A study in the US that reviewed more than 2000 prescriptions issued by doctors in various training programmes revealed that the error rate for those in a family medicine training programme was 11%.^14^ It is encouraging that the self-review process was able to yield comparable error rates, although it would be useful to determine whether the same sort of errors are being reported.

There is evidence to suggest that GP trainees may lack a systematic and comprehensive education in safe prescribing.^6,7^ Feedback has been shown to reduce prescribing errors among doctors-in-training and postgraduate doctors in secondary care,^15,16^ with Ferguson *et al* ^17^ concluding that effective feedback is timely and provides a benchmark against which a participant can compare their prescribing performance. Although health care workers (physicians) may not accurately assess their own performance,^18^ Van der Steen *et al* ^19^ recently reported that self-assessment of prescribing by junior doctors significantly reduced potentially harmful prescriptions in secondary care.

### Implications for research and practice

In July 2020, the prescribing assessment was approved by the GMC and was rolled out as a ‘formal’ prescribing assessment for the 2020–2021 cohort of GP trainees in England.^20^ The main concern regarding the assessment had been the amount of time that the review process had taken the trainees to perform. The 2020–2021 and subsequent cohorts were therefore tasked with reviewing 50 prescriptions rather than 60. For this first cohort of reduced prescription scrutiny, the percentage of trainees taking >4 hours fell from 63.2% to 52.5%, with no discernible reduction in assessment validity, or participant satisfaction.^21^ The prescribing assessment has, to date, now been completed by more than 5900 GP trainees in the UK. Although the assessment is valued by trainer and trainee, further research should investigate whether undertaking the process actually reduces rates of prescription error. It is also useful to consider how the assessment could be augmented. Brown^22^ and colleagues have reported encouraging findings regarding pharmacist involvement in the process, which merit further consideration.

## Data

The data can be accessed by contacting the authors.
